# Key person-centered care domains for residential substance use disorder treatment facilities: former clients’ perspectives

**DOI:** 10.1186/s13011-023-00554-x

**Published:** 2023-07-17

**Authors:** Barbara Andraka-Christou, Danielle N. Atkins, Morgan C. Shields, Olivia K. Golan, Rachel Totaram, Kendall Cortelyou, Glenn W. Lambie, Olena Mazurenko

**Affiliations:** 1 School of Global Health Management & Informatics, University of Central Florida, 525 W Livingston Street, Suite 401, Orlando, FL 32801 USA; 2 Department of Internal Medicine, University of Central Florida, Orlando, FL USA; 3 Askew School of Public Administration, Florida State University, Tallahassee, FL USA; 4 Brown School, Washington University in St. Louis, St. Louis, United States; 5 School of Public Health, Georgia State University, Atlanta, Georgia; 6 Department of Counselor Education & School Psychology, University of Central Florida, Orlando, FL USA; 7 Department of Health Policy & Management, Fairbanks School of Public Health, Indiana University, Indianapolis, IN USA

**Keywords:** Residential, Substance use disorder, Person-centered care, Preferences, Survey, Social media, Patient-centered care

## Abstract

**Background:**

While person-centered care (PCC) includes multiple domains, residential substance use disorder (SUD) treatment clients may value certain domains over others. We sought to identify the PCC domains most valued by former residential SUD treatment clients. We also sought to explore conceptual distinctions between potential theoretical PCC subdomains.

**Methods:**

We distributed an online survey via social media to a national convenience sample of former residential SUD treatment clients. Respondents were presented with ten PCC domains in an online survey: (a) access to evidence-based care; (b) integration of care; (c) diversity/respect for other cultures; (d) individualization of care; (e) emotional support; (f) family involvement in treatment; (g) transitional services; (h) aftercare; (i) physical comfort; and (j) information provision. Respondents were asked to select up to two domains they deemed most important to their residential SUD treatment experience. We used descriptive statistics to identify response frequencies and logistic regression to predict relationships between selected domains and respondents’ race, gender, relationship status, parenting status, and housing stability.

**Results:**

Our final sample included 435 former residential SUD treatment clients. Diversity and respect for different cultures was the most frequently selected domain (29%), followed by integration of care (26%), emotional support (26%), and individualization of care (26%). Provision of information was the least frequently chosen domain (3%). Race and ethnicity were not predictive of selecting respect for diversity. Also, parental status, relationship status and gender were not predictive of selecting family integration. Employment and housing status were not predictive of selecting transitional services.

**Conclusions:**

While residential SUD treatment facilities should seek to implement PCC across all domains, our results suggest facilities should prioritize (a) operationalizing diversity, (b) integration of care, and (c) emotional support. Significant heterogeneity exists regarding PCC domains deemed most important to clients. PCC domains valued by clients cannot be easily predicted based on client demographics.

**Supplementary Information:**

The online version contains supplementary material available at 10.1186/s13011-023-00554-x.

## Background

The United States (US) is experiencing multiple substance use disorder (SUD) crises with rising rates of opioid and stimulant overdoses [1]. Residential facilities, which provide short or long-term (i.e., longer than 30 days) care in non-hospital settings, are common environments for SUD treatment [2]. More than 1,400 non-federal residential SUD treatment facilities exist in the US [3]. Among Americans aged 12 or older who received SUD treatment in 2020, 27.5% received treatment in a residential facility [2]. Residential treatment is costly, ranging from $5,700 to $17,000 per client per treatment episode [3]. As compared to clients receiving SUD treatment in the outpatient setting, clients receiving SUD treatment in residential facilities tend to have more complex needs [4]. Unfortunately, little is known about the quality of care provided in residential facilities [5].

Person-centered care (PCC) is a key quality indicator [6, 7] and is associated with several healthcare outcomes (e.g., treatment retention, safety) [8, 9], including in residential SUD facilities. PCC postulates putting individual as a key decision-maker in care and holistically addressing unique patient care needs and preferences [10]. Numerous conceptual PCC frameworks exist outlining several key elements of care delivery [10–13]. Importantly, the Picker Institute’s PCC framework is among the most commonly used, having been adopted by the Institute of Medicine [6] and informing quality metrics used by the US National Committee for Quality Assurance, as part of health plan accreditation and by the Centers for Medicaid and Medicare Services for value based programs [14]. According to the Picker framework, PCC is primarily defined by the patient experience, rather than patient satisfaction, and consists of eight domains [15, 16], which were later described as dimensions or domains by the Institute of Medicine: [1] respect for patient preferences, values, and culture; [2] provision of education/information; [3] integration of care; [4] provision of emotional comfort; [5] provision of physical comfort; [6] integration of family; [7] facilitation of transition into the community; and [8] provision of access to evidence-based care. All eight domains are considered necessary and are predictive of positive healthcare outcomes [8]. Our previous work identifies that some PCC domains may be further broken into subdomains [17]. For example, within the domain of “respect for patient preferences, values, and cultures,” our team has previously found suggestive qualitative evidence that subdomains might include (a) diversity and respect for other cultures (e.g., having staff from similar racial/ethnic backgrounds to those of clients), and (b) individualization of care (e.g., permitting clients to select their own treatment or set their own goals). Similarly, we have suggestive qualitative evidence that the domain “transition and continuity of care” includes the conceptually distinct subdomains of (a) transition into the community (e.g., assistance with securing housing or employment after residential treatment) and (b) aftercare (e.g., wellness check-ups; continued but less intensive treatment).

While the literature on PCC theory is extensive [18], little is known about PCC domains for residential treatment of SUD. Efforts to improve PCC in residential SUD facilities are critically important, because SUD treatment in the US has a history of paternalistic and coercive practices [19]. Furthermore, recent research findings identify underutilization of evidence-based treatment in residential facilitates [20, 21], and minimal permitted contact between the client and their family/community [5, 22, 23]. For example, to encourage clients to focus on their recovery, it is not uncommon for residential SUD facilities to limit excursions into the community (e.g., to visit family), especially during the beginning of treatment [24–26], potentially heightening the risk of clients experiencing coercive practices. People receiving SUD treatment are also a particularly vulnerable population, often having experienced systematic stigmatization (e.g., due to drug criminalization) [27, 28], and may feel low empowerment when seeking or receiving treatment [29, 30]. A nationally representative study of SUD treatment facilities in the US, focusing on the PCC “respect” domain, found that fewer than half of residential facilities (39%) invited patients to participate in clinical decision-making processes [31]. Despite the unique constraints on PCC present in residential settings (e.g., physical separation from social supports) [22–24], most studies of PCC in SUD treatment focus on outpatient settings [10] where the risk of coercive practices is arguably lower. Further, some PCC domains are particularly understudied in residential SUD treatment, such as provision of physical comfort and integration of family into treatment [32].

While residential SUD treatment facilities should fully implement PCC across all eight domains, resource-limited facilities engaging in quality improvement efforts may need to focus on one or two domains at a time, and prioritization decisions should be informed by client preferences. To our knowledge, no previous study has examined how residential SUD treatment clients value PCC domains (e.g., whether they believe certain domains are more important than others). Therefore, to inform residential SUD treatment facilities’ quality improvement efforts, we sought to identify the PCC domains deemed most important to clients and to explore whether our hypothesized PCC subdomains are conceptually distinct. We used a social media survey of a national convenience sample of former residential SUD treatment clients.

We hypothesized that respondents who select respect for diversity and culture would not necessarily select individualization of care, and we hypothesized that respondents who select transitional services would not select aftercare. We hypothesized that certain respondent characteristics would be associated with selection of certain PCC domains. As compared to their counterparts, we hypothesized that respondents who were women, people in a relationship with a significant other, and/or parents would be more likely to prioritize “family involvement in treatment.” As compared to white or non-Hispanic respondents, we hypothesized that Black or Hispanic respondents would be more likely to prioritize “diversity and respect for different cultures.” Compared to stably housed respondents, we hypothesized that respondents experiencing housing instability would be more likely to prioritize “transitional services.” Finally, we hypothesized that unemployed respondents seeking work would be more likely to prioritize “transitional services” compared to other respondents.

## Methods

### Instrument development

Our research team drafted a survey instrument informed by the Institute of Medicine’s definition of PCC [15, 33], as well as interviews with residential and outpatient SUD treatment clients and staff from an earlier study [17]. The instrument asked respondents to select up to two PCC domains they consider most important for residential SUD treatment, with examples of PCC experiences informed by our prior work [34]. PCC response options on the survey were as follows: [1] access to evidence-based treatments for addiction (for example, counseling, addiction medications); [2] integration of care (for example, offering physical and/or mental health screenings and care on-site or off-site); [3] diversity and respect for different cultures (for example, racially/ethnically diverse staff, bilingual staff, services specifically for lesbian, gay, bisexual, transgender, or queer people); [4] individualization of care (for example, choice about the type of treatment used); [5] emotional support (for example, compassionate staff, peer recovery support specialists); [6] family involvement in treatment; [7] transitional services (for example, job training/application assistance, help with housing); [8] aftercare (for example, wellness checks after discharge); [9] physical comfort (for example, clean facilities, comfortable bedding, roommate choice); and [10] information (for example, information about the purpose and types of treatment). Respondents could also select “none of the above are important.”

Based on our prior qualitative work [34], for the survey instrument, we broke the original domain “respect for client preferences, values, and culture” into two domains: “diversity and respect for different cultures” and “individualization of care.” Similarly, for our survey instrument, we broke the original domain “transition into the community” into two domains: “transitional services” and “aftercare.” See Table 1 for a list of domains originally theorized by Gerteis et al. (1993), as well as our newly created domains.

**Table 1:** New and original domains

Original PCC domains (*n* = 8)	Our PCC domains (*n* = 10)
Access to evidence-based treatments	Access to evidence-based treatments
Integration of care	Integration of care
Respect for patient values, preferences, and needs	Diversity and respect for other cultures
	Individualization of care
Emotional support	
Family involvement in treatment	
Transition and continuity of care	Transitional services
	Aftercare
Physical comfort	Physical comfort
Provision of information	Provision of information

*Note: Highlighted rows indicate divergence of domains tested in our study from original domains by Gerteis et al. (1993)*

We asked for selection of up to two most important PCC domains rather than ranking all domains to minimize cognitive load. Based on our qualitative work, we had suggestive evidence that all domains are considered important to a certain extent. Still, we lacked information on which PCC domains were considered the *most* important. Therefore, we avoided a Likert scale in order to prevent respondents from simply agreeing that each domain was important (i.e., selecting a 5 for each domain).

Additionally, the survey instrument included several demographic questions, including respondent race, ethnicity, gender^1^, housing status, employment status, parental status, and relationship status. See Appendix A for the instrument. The survey instrument was piloted with six former clients of residential SUD treatment facilities. During the piloting process, clients recommended providing brand names of medications, expanding the list of potential staff roles, providing additional examples of transitional services needed by clients, explicitly defining certain terms, and decreasing the number of response options per question to minimize cognitive load. The refined version of the survey was imported into Qualtrics software.

### Ethics

The first author’s university’s Institutional Review Board provided approval and ethical oversight of our research. Survey respondents were provided with an explanation of the research prior to data collection. Verbal or written consent was not collected from respondents.

### Data collection

Our inclusion criteria for survey respondents were speaking English, being 18 years of age or older, and having experienced residential SUD treatment. We used Facebook to recruit survey respondents. We posted a recruitment message with the survey link to Facebook groups related to SUD, such as recovery groups. We offered a $10 electronic gift card to compensate respondents for their time.

### Data analysis

We started with 581 respondents but excluded observations based on several approaches to improve data quality for a final sample of 435 respondents. First, we excluded respondents who did not finish the survey (*n* = 9) and those Qualtrics identified as spam (*n* = 2). Then we excluded respondents who responded that they were less than 18 years of age (*n* = 4) and those who said they had experienced zero residential treatment episodes (*n* = 41). Next, we excluded respondents whose IP addresses were identified as server farms using the STATA package “checkipaddresses,” which traces and scores IP addresses to identify server farms (*n* = 16) [35]. In line with prior literature, we also excluded respondents who completed the survey in less than two minutes (*n* = 4) and those who incorrectly answered factual attention check questions (e.g., “Which of the following words is NOT an animal?”) (*n* = 20). We also excluded those who indicated they did not learn about the survey through social media after we posted the survey on social media (*n* = 12). We excluded respondents with missing responses and those who selected “choose not to answer” for variables included in this analysis (*n* = 38) for a total of 435 responses remaining.

Our primary outcomes of interest were the two PCC domains selected as “most important” out of ten potential options. We created dichotomous indicator variables equal to 1 if the respondent selected the domain and 0 otherwise. We calculated descriptive statistics showing the percentage of respondents who selected each PCC domain. We also examined the percentage of respondents who selected both domains.

We also estimated descriptive statistics for the covariates of interest, including respondent sociodemographic characteristics such as gender, race, age, employment status, and significant other. Employment status was coded as dichotomous indicator variables for those who reported they were employed, out of work and looking for work, or other employment (e.g., homemaker, student, disabled). Significant other was a dichotomous indicator variable equal to 1 if the respondent reported being married or not married but in a relationship with a significant other and 0 otherwise. Stable housing was an indicator variable equal to 1 if the respondent reported living in a house or an apartment and 0 otherwise (e.g., living in shelter, on the street, or in transitional housing). Multiple logistic regressions were estimated to explore associations between respondent sociodemographic characteristics and the likelihood they selected a domain, with results reported as odds ratios. All analyses were conducted in STATA 16.

## Results

Our final sample consisted of 435 respondents. Table 2 shows descriptive statistics for the sample. The average age was 34 years, with approximately half of the sample being male (53%). Most respondents were non-Hispanic White (74%), employed (72%), parents (64%), stably housed (70%; i.e., they lived in a house or an apartment), rated their health over the four weeks before the survey as either very good or excellent (69%), and had a significant other (84%; i.e., they were married or in a relationship).

**Table 2:** Respondent characteristics (*N* = 435)

	*M*	*N*
Age	34.35	
**Gender**		
Male	53.3%	232
Female	46.7%	203
**Race/ethnicity**		
Black	12.4%	54
White	74.0%	322
Hispanic	10.6%	47
Other race	1.8%	8
More than one race	0.9%	4
**Self-assessed health**		
Very good/excellent health	69.1%	300
Fair/poor/very poor health	30.9%	135
**Relationships**		
Parent	63.7%	277
Significant other	83.9%	365
**Employment**		
Employed	72%	313
Out of work and looking for work	11.7%	51
Employment - other	16.3%	71
**Housing**		
Stable housing	70.1%	305
Unstably Housed	29.9%	130
Observations	435	

**Fig. 1:**
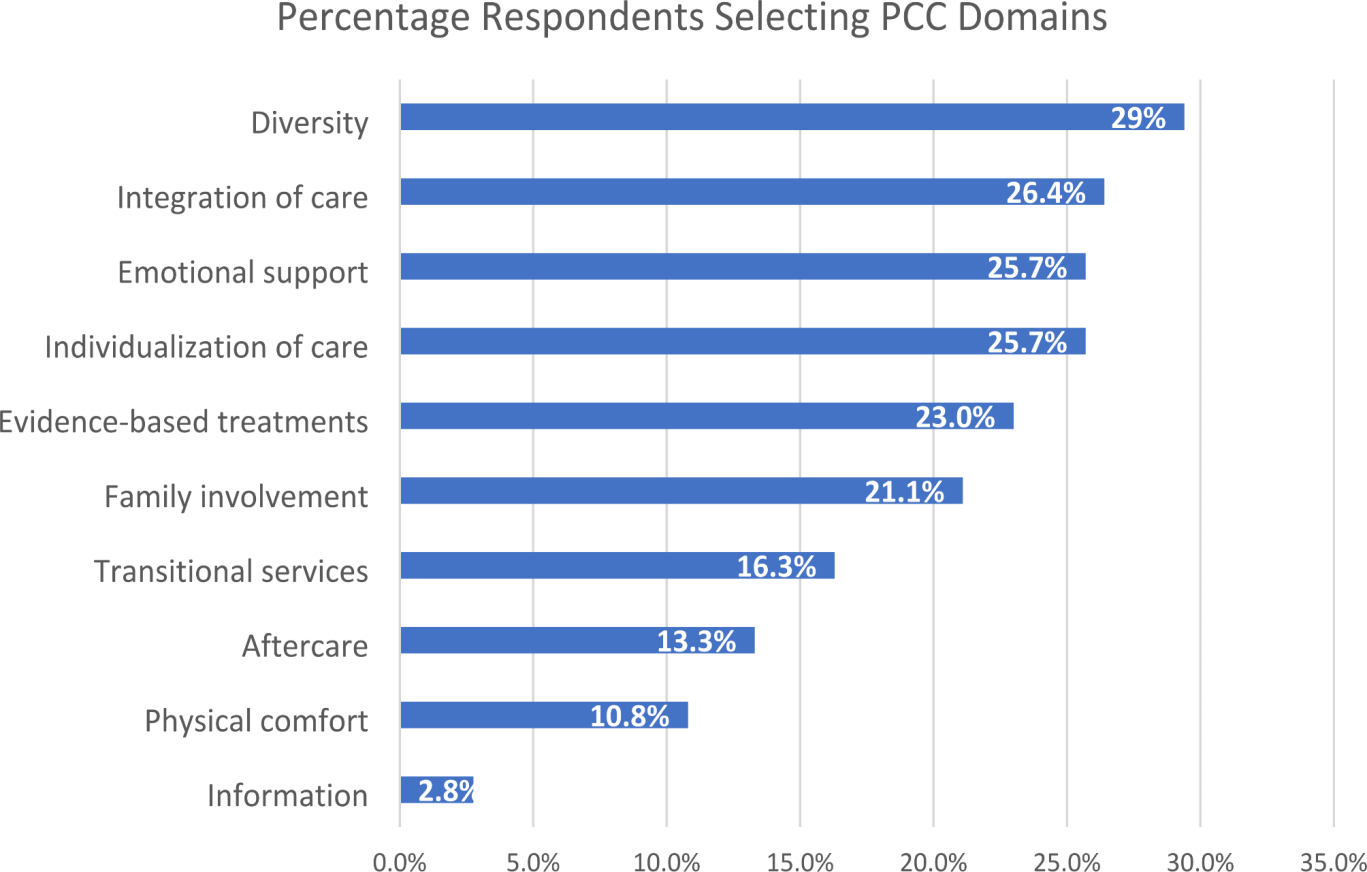
Percentage of respondents selecting each PCC domain as “most important” (n = 435)

Figure 1 shows the percentage of respondents selecting each of the 10 PCC domains. These domains are not mutually exclusive categories, because respondents could select up to two domains. Diversity and respect for different cultures was the most frequently selected domain (29%), followed by integration of care (26%), emotional support (26%), individualization of care (26%), access to evidence-based treatments (23%), and family involvement in treatment (21%). Transitional services were less commonly selected (16%), as were aftercare (13%) and physical comfort (11%). Information sharing (e.g., about the purpose of treatment) was the least commonly selected domain (3%).

Table 3 shows the percentage of respondents who chose each pair of domains. Some respondents selected only one domain (~ 5%), so these percentages do not add up to 100. We hypothesized that respondents who select respect for diversity and culture would not necessarily select individualization of care, and we hypothesized that respondents who select transitional services would not select aftercare. The most commonly selected pairs were “diversity and respect for different cultures” and “family involvement in treatment” (8%); followed by “diversity and respect for different cultures” and “emotional support” (6%); “access to evidence-based treatment” and “individualization of care” (6%); and “access to evidence-based treatment” and “integration of care” (5%). Several of the domain-pairs were selected by fewer than 1% of respondents.

**Table 3:** Frequency with which respondents selected two domains

Variables	Evidence-based treatments	Integration of care	Diversity	Individualization of care	Emotional support	Family involvement	Transitional services	Aftercare	Physical comfort
Evidence-based treatments									
Integration of care	5.06%								
Diversity	3.22%	2.99%							
Individualization of care	5.75%	4.60%	2.53%						
Emotional support	2.99%	4.60%	6.21%	1.15%					
Family involvement	3.22%	2.07%	7.59%	2.76%	1.61%				
Transitional services	0.69%	2.07%	2.99%	4.60%	2.76%	0.23%			
Aftercare	0.92%	1.84%	1.38%	2.53%	2.53%	1.61%	0.92%		
Physical comfort	0.46%	1.38%	1.61%	0.69%	2.99%	1.38%	1.15%	0.92%	
Information	0.00%	0.00%	0.00%	0.23%	0.92%	0.46%	0.69%	0.46%	0.00%

We hypothesized that respondents who were women, people in a relationship with a significant other, and/or parents would be more likely to select “family involvement in treatment.” As compared to white or non-Hispanic respondents, we hypothesized that Black or Hispanic respondents would be more likely to prioritize “diversity and respect for different cultures.” Compared to stably housed respondents, we hypothesized that respondents experiencing housing instability would be more likely to prioritize “transitional services.” Finally, we hypothesized that unemployed respondents seeking work would be more likely to prioritize “transitional services” compared to other respondents.

Table 4 shows the results from logistic regression models examining the relationship between respondent sociodemographic characteristics and the likelihood of selecting each PCC domain. Results are reported as odds ratios. Although none of our hypotheses were supported, some demographics were associated with selecting certain domains. We found that White individuals (OR 0.243; *p* < 0.001) and those with significant others (OR 0.385; *p* < 0.01) were less likely to select evidence-based treatments while those out of work and looking for work (OR 4.327; *p* < 0.001) and those who were stably housed (OR 2.492; *p* < 0.01) were more likely to select evidence-based treatments. We found that parents were more likely to select integration of care (OR 1.952; *p* < 0.05). Individuals with significant others were more likely to select diversity as a top domain than those without significant others (OR 2.334; *p* < 0.05). Those who were out of work and looking for work (OR 2.617, *p* < 0.01) and parents (OR 2.082; *p* < 0.01) were more likely to select individualization of care. Older individuals were more likely to select emotional support (OR 1.038; *p* < 0.05), while those who were out of work and looking for work (OR 0.164; *p* < 0.01) and parents (OR 0.516; *p* < 0.05) were less likely to select emotional support. Older individuals were more likely to select aftercare (OR 1.089; *p* < 0.001). White respondents were more likely to select physical comfort (OR 2.775; *p* < 0.05) while parents were less likely (OR 0.474; *p* < 0.05) to select physical comfort. Finally, older respondents were more likely to select information as a top domain (OR 1.157; *p* < 0.01) while those with significant others were less likely to select information as a top domain (OR 0.0239; *p* < 0.05).

**Table 4:** Odds ratios for likelihood of selecting domain

	(1)	(2)	(3)	(4)	(5)	(6)	(7)	(8)	(9)	(10)
	Evidence-based treatments	Integration of care	Diversity	Individualization of care	Emotional support	Family involvement	Transitional services	Aftercare	Physical comfort	Information
Age	1.031	0.963	0.972	0.964	1.038*	1.002	1.029	1.089***	0.983	1.157**
	[0.988,1.075]	[0.928,1.000]	[0.937,1.009]	[0.928,1.003]	[1.000,1.077]	[0.964,1.043]	[0.986,1.074]	[1.039,1.140]	[0.930,1.039]	[1.051,1.273]
Female	1.033	0.885	1.023	1.041	0.988	1.071	0.846	0.814	1.440	0.658
	[0.621,1.719]	[0.569,1.378]	[0.669,1.564]	[0.662,1.636]	[0.632,1.545]	[0.667,1.719]	[0.502,1.427]	[0.456,1.452]	[0.768,2.702]	[0.188,2.308]
White	0.243***	0.639	1.112	1.209	1.108	1.781	1.160	2.097	2.775*	4.581
	[0.143,0.413]	[0.387,1.057]	[0.669,1.849]	[0.703,2.079]	[0.645,1.905]	[0.963,3.295]	[0.616,2.182]	[0.930,4.726]	[1.101,6.997]	[0.536,39.172]
Out of work and looking for work	4.327***	0.712	0.862	2.617**	0.164**	0.857	0.698	0.561	0.992	1.904
	[2.168,8.635]	[0.347,1.463]	[0.427,1.742]	[1.346,5.086]	[0.049,0.549]	[0.387,1.899]	[0.276,1.766]	[0.186,1.694]	[0.320,3.079]	[0.362,10.011]
Significant other	0.385**	0.742	2.334*	1.781	1.471	0.579	1.230	0.907	0.922	0.239*
	[0.191,0.775]	[0.382,1.444]	[1.169,4.661]	[0.804,3.948]	[0.752,2.875]	[0.295,1.136]	[0.556,2.722]	[0.400,2.057]	[0.400,2.127]	[0.060,0.947]
Parent	0.796	1.952*	0.821	2.082**	0.516*	1.445	0.783	0.672	0.474*	0.445
	[0.436,1.454]	[1.124,3.390]	[0.505,1.338]	[1.188,3.651]	[0.310,0.858]	[0.807,2.586]	[0.431,1.423]	[0.345,1.306]	[0.234,0.958]	[0.107,1.852]
Stable housing	2.492**	0.702	0.754	1.327	1.144	0.777	1.226	0.801	1.588	1.003
	[1.321,4.702]	[0.435,1.132]	[0.475,1.197]	[0.797,2.211]	[0.693,1.890]	[0.469,1.285]	[0.682,2.204]	[0.436,1.472]	[0.759,3.323]	[0.277,3.629]
Observations	435	435	435	435	435	435	435	435	435	435

Notes: Exponentiated coefficients; confidence intervals in brackets

## Discussion

This is the first study to explore which PCC domains are most valued by former residential SUD treatment clients. We found that a national convenience sample of former residential SUD treatment clients were most likely to select “diversity and respect for different cultures” as important and least likely to select “provision of treatment information” as important, among eight other PCC domain options. Despite these trends, we found significant heterogeneity in PCC domains deemed most important by former clients. Furthermore, none of our hypotheses regarding the associations between sociodemographic characteristics and PCC domains deemed important were supported.

Former residential SUD treatment clients were somewhat more likely to select “respect for diversity and other cultures” as important, as compared to other PCC domains. Even though residential SUD facilities should seek to operationalize all PCC domains, resource-constrained residential SUD facilities could focus on operationalizing this domain in the short-term. One approach to operationalizing diversity and respect for different cultures is evaluating and responding to the needs of culturally diverse treatment populations, sometimes called “culturally competent treatment.” Culturally competent treatment practices include the following: (a) language congruence between staff and clients; (b) cross-cultural training about needs, preferences, and beliefs of specific minoritized groups; (c) racial/ethnic minority representation among staff and management; (d) assessing cultural/religious preferences of clients; providing specific programming for lesbian, gay, bisexual, and transgender clients (LGBT); and (e) allowing transgender clients to sleep in the dormitory of the gender with which they identify [34, 36]. Existing studies suggest that women, people of racially minoritized background/ethnicities, and lesbian, gay, bisexual, and transgender people may have unique SUD treatment needs [37–39]. For example, women are more likely than men to have eating disorders and a history of sexual trauma, potentially necessitating women-only counseling groups for women to feel comfortable while discussing their behavioral health history [37].

A recent meta-analysis of culturally sensitive treatment for racially/ethnically minoritized youth found greater reductions in post-treatment substance use as compared to controls [40]. A separate study found that organizations that adopted culturally competent practices had reduced wait times for and longer retention of Black and Hispanic clients [36]. Unfortunately, prior studies suggest culturally competent practices may be uncommon in SUD facilities [36, 41, 42]. For example, only 18% of SUD treatment facilities in a recent national study reported providing LGBT-specific programming [43].

Importantly, none of our other hypotheses regarding associations between PCC domains selected as most important and respondent demographic characteristics were supported. For example, we did not see associations between parenting or significant other status and selection of family integration, nor did we see associations between employment or housing status and selection of transitional services. Similarly, respondent race and ethnicity did not predict selection of respect for diversity and other cultures. Our results suggest that researchers, clinicians, and facility administrators cannot assume PCC domains deemed important to specific clients merely based on the clients’ sociodemographic characteristics. Significant heterogeneity in the PCC domain deemed most important to clients is also indicated by the narrow difference in proportions of respondents who selected each of the top four domains. In other words, it appears that PCC domain preferences are highly individualized, with no clear PCC domain preference across clients, and PCC domain preferences are difficult to predict at the client level. Facilities could consider discussing PCC preferences with clients before beginning treatment and then modify treatment plans and ancillary services accordingly. For example, clients for whom family integration is a priority can have extended visitation times with family members.

Furthermore, we did not find any clear trends concerning the selection of pairs of top two domains. More than 8% of people selected no possible pair of priorities, further suggesting heterogeneity of treatment priorities across our sample. This result also provides preliminary, suggestive evidence of conceptual distinctions between potential subdomains of “respect for diversity and other cultures” and “individualization of care,” as well as between “transitional services” and “aftercare.”

Our study has several important limitations. First, we used a convenience sample, which limits the generalizability of results. For example, individuals recruited via social media have access to technology and the Internet and, therefore, may have different socioeconomic characteristics from the typical former residential treatment client. As compared to demographic characteristics in a national study of residential clients from 2011, respondents in our study were more likely to be white, female, stably housed, and employed [44]. Second, the results for the logistic regressions are correlational and causal inferences cannot be drawn. Third, we did not assess the amount of time that has passed since respondents left residential treatment. Memories of residential treatment may change over time, which may influence preferences. Fourth, we could not distinguish by respondent type of SUD, even though PCC priorities may differ based on SUD type. Finally, it is possible that wording of examples for each domain biased the selection of the domain. For example, for the aftercare subdomain, we provided the example “wellness checks after discharge,” but we did not provide the example “outpatient treatment.” Similarly, even though the instrument was piloted, it was not validated; therefore, it is possible that heterogeneity existed in respondents’ interpretations of the meanings of certain domains. For example, our hypothesis regarding the association between respondent race/ethnicity and selection of “respect for diversity and other cultures” was even less likely to be supported if respondents interpreted this domain to include respect for personal characteristics beyond race/ethnicity (e.g., sexual orientation, gender, ability, etc.) We also did not include all possible housing arrangements, such as renting a house (as compared to an apartment), which could have impacted how respondents selected housing arrangements, potentially impacting our findings.

## Conclusions

A national convenience sample of former clients in residential SUD treatment recruited via social media were most likely to select “diversity and respect for different cultures” as an important PCC domain as compared to nine of the potential options. Therefore, while residential SUD treatment facilities should seek to implement all PCC domains, in the short-term fully, they could prioritize efforts at improving “diversity and respect for different cultures.” Furthermore, none of our hypotheses regarding respondent sociodemographic characteristics and PCC domain selection were supported, suggesting that PCC preferences are highly individual. Results of this pilot study could inform the development of future instruments measuring PCC preferences with evidence of validity and reliability of the scores.

## Electronic supplementary material

Below is the link to the electronic supplementary material.

**Additional Files** File name: Appendix A Social Media PCC paper. Title of data: Appendix A Social Media PCC Paper. Description of data: Relevant Questions from Social Media Paper Instrument.

## Data Availability

Deidentified data and materials are available upon request to corresponding author and following appropriate institutional review board approval.

## List of abbreviations

LGBT: Lesbian, gay, bisexual, transgender
PCC: Person-centered care
SUD: Substance use disorder

## Footnotes

1 The survey included responses for male, female, other, and choose not to answer, but the final sample only included respondents who selected male or female based on other exclusion criteria.
