# Supplementary material for: Key person-centered care domains for residential substance use disorder treatment facilities: former clients’ perspectives
Source: Subst Abuse Treat Prev Policy. 2023 Jul 17;18:45. doi: 10.1186/s13011-023-00554-x (PMC10353234; doi:10.1186/s13011-023-00554-x)
Supplement: Supplementary file 1 — Additional Files File name: Appendix A Social Media PCC paper. Title of data: Appendix A Social Media PCC Paper. Description of data: Relevant Questions from Social Media Paper Instrument. [file 13011_2023_554_MOESM1_ESM.docx]

**APPENDIX A: Relevant questions from the PCC survey**

Of the following, which two are most important for a residential addiction treatment facility to offer? Select **up to two** based on your preferences.

- Access to evidence-based treatments for addiction (for example, counseling, addiction medications)
- Integration of Care (for example, offering physical and/or mental health screenings and care on-site or off-site)
- Diversity and respect for different cultures (for example, racially/ethnically diverse staff, bilingual staff, services specifically for lesbian, gay, bisexual, transgender, or queer)
- Individualization of care (for example, choice about the type of treatment used)
- Emotional support (for example, compassionate staff, peer recovery support specialists)
- Family involvement in treatment
- Transitional services (for example, job training/application assistance, help with housing)
- Aftercare (for example, wellness checks after discharge)
- Physical Comfort (for example, clean facilities, comfortable bedding, roommate choice)
- Information (for example, information about the purpose and types of treatment)
- None of the above are important

What is your gender?

- Male
- Female
- Other
- Choose not to answer

What is your race/ethnicity? Mark all that apply.

- African American/Black
- Asian
- Pacific Islander/Native American
- White
- Hispanic
- Other
- Choose not to answer

What is your current source(s) of income/support? Mark all that apply.

- Wages/salary
- Public assistance
- Retirement/pension
- Disability
- None
- Choose not to answer

What is your age? Please enter a number below.

Overall, how would you rate your health in the last 4 weeks?

- Excellent
- Very good
- Fair
- Poor
- Very poor
- Choose not to answer

In the **last week**, which of the following best describes where you have slept overnight?

- A shelter
- Outside, on the street, or in an abandoned building
- Transitional housing
- Couchsurfing or between homes
- House that you own
- Apartment
- Hotel room
- Other______________________________________________
- Choose not to answer

What is your current employment status?

- Employed for wages
- Self-employed
- Out of work and looking for work
- Out of work but not currently looking for work
- A homemaker
- A student
- Military
- Retired
- Unable to work/disabled
- Choose not to answer

What is your current relationship status?

- Single
- Married
- Not married but in a relationship with a significant other
- Widowed
- Other
- Choose not to answer

Are you a parent?

- Yes
- No
- Choose not to answer
